# Full-Factorial Rheological Investigation of Carbopol ETD2020 for Embedded Printing: Effects of pH and Carbomer Concentration

**DOI:** 10.3390/ma18133164

**Published:** 2025-07-03

**Authors:** Tobias Biermann, Lennart Mesecke, Simon Teves, Ivo Ziesche, Roland Lachmayer

**Affiliations:** 1 Institute of Product Development, Leibniz University Hannover, An der Universität 1, 30823 Garbsen, Germany; mesecke@ipeg.uni-hannover.de (L.M.); teves@ipeg.uni-hannover.de (S.T.); lachmayer@ipeg.uni-hannover.de (R.L.); 2 Cluster of Excellence PhoenixD, Leibniz University Hannover, Welfengarten 1A, 30167 Hannover, Germany

**Keywords:** Carbopol ETD2020, embedded printing, rheological design window, shear-thinning hydrogel, carbomer pH adjustment, Herschel–Bulkley modeling

## Abstract

Embedded printing of soft materials relies on yield-stress support matrices to prevent sagging and enable freeform fabrication. The rheological parameters of the matrix material directly influence critical printing outcomes such as strand positioning, cavity formation, structural stability, and defect suppression in embedded printing. Despite widespread use of *Carbopol^®^* formulations, a systematic rheological characterization of *ETD2020* across relevant polymer concentrations and pH levels for embedded printing is lacking. Here, we implement a full-factorial design with polymer concentrations from 0.1wt% to 0.9wt% and triethanolamine dosages of 30–50µL per 100g. Steady-shear (0.001–$200\;s^{-1}$) and oscillatory (1Hz) rheometry yielded Herschel–Bulkley parameters $\tau_{y}$, *k*, *n* as well as storage and loss modulus $G^{\prime }$/$G^{\prime \prime }$. All formulations exhibited pronounced shear-thinning, with $\tau_{y}$ increasing nonlinearly from <1Pa to 41.1Pa and $G^{\prime }$ reaching ≈400Pa at 0.9wt%. A five-hour window of invariant rheology was identified, followed by a $\Delta \tau_{y}\approx 10\;\mathrm{Pa}$ increase after five days, indicating delayed polymerization. The comprehensive material characterization defines a rheological window for *ETD2020* and facilitates simulation-based modeling and the targeted tuning of matrix properties. Heatmaps provide an interpolated depiction of combined carbomer and triethanolamine concentrations, enabling tunable support matrices for embedded printing.

## 1. Introduction

Additive manufacturing with silicone-based materials has emerged as a key enabling technology for the fabrication of soft, functional components. Due to their high flexibility, chemical stability, and optical transparency, silicone elastomers are used in soft robotics [1,2,3], biomedical devices [4,5,6], microfluidic networks [7,8], as well as optical applications [9].

Embedded printing has gained particular relevance for the processing of soft and slow-curing materials [10]. By extruding the ink directly into a yield-stress support matrix, this approach overcomes gravitational sagging and enables the freeform fabrication of intricate 3D structures without the need for external supports [11,12,13]. The process is especially suitable for silicone that shows a long pot life and a high viscosity [3,14]. As the morphology and stability of printed structures strongly depend on the interplay between the extruded material and the surrounding matrix, understanding and tailoring the rheological behavior of the support material is critical for successful printing.

As Herschel–Bulkley fluids, suitable matrix systems must simultaneously provide structural support at rest and allow localized displacement under shear during extrusion [7,15,16,17]. The key model parameters yield stress ($\tau_{y}$), consistency index (*k*), and flow index (*n*) govern the flow behavior across the relevant shear rate regimes and serve as primary descriptors of the printability [18,19].

The rheological parameters of the matrix material govern several critical effects in embedded printing. A high viscosity ratio $\eta^{*}$ between ink and matrix material directly reduces the vertical *z*-offset Δz of the printed strand [18]. The extent of the locally liquefied zone around the nozzle tip as well as the resulting material displacement behavior is determined by the matrix’s yield stress $\tau_{y}$, flow index *n*, and consistency index *k*, as well as the nozzle diameter $d_{a}$ and print speed $v_{V}$, and is described via the Oldroyd number Od [14]. Cavity formation behind the moving nozzle results from the interplay between hydrostatic pressure and viscous stresses in the matrix [15]; if the hydrostatic pressure at the rear of the cavity does not exceed $\tau_{y}$, static cavities remain [13]. Undesired necking and rupture of printed strands are attributed to mismatches in interfacial energy and $\tau_{y}$, influenced by the strand radius [13,16]. Additionally, rupture is favored when the storage modulus of the ink is small relative to the matrix [14]. No general link between filament fusion and matrix rheology has been formulated. Only Grosskopf et al. report successful merging in crossing paths, whereas parallel paths tend to shift previously deposited material. This phenomenon decreases with increasing Od [14]. Theoretically, sagging and thixotropic instabilities could occur [13,15,16], yet sagging is negligible due to minimal density differences between most ink and matrix materials, and thixotropic effects are usually irrelevant for Carbopol matrices with recovery times <1 s [12,15,19].

*Carbopol^®^ Carbomers* are frequently used as a support matrix in embedded printing due to their high transparency, biocompatibility, and tunable rheological profile [7,12,13,15,20]. The *Carbopol^®^ ETD2020* formulation allows for wide-ranging adjustment of mechanical properties through controlled variation of polymer concentration and neutralization level, making it particularly attractive for applications requiring precise extrusion stability and reproducible feature resolution [12,13,20]. While *Carbopol^®^*-based support matrices such as *Carbopol^®^ 940*, *Carbopol^®^ 980-NF* and *Ultrez 10* have been widely explored [12,21], a systematic rheological characterization of *ETD2020* across a formulation space with relevant parameters for embedded printing is not available. Previous studies typically rely on a few discrete compositions [1,15,22], address compositions not relevant for embedded printing [23] or underestimate the role of pH in determining key flow parameters such as $\tau_{y}$, *k*, *n* [1,22,24,25,26]. To address these gaps, the present study applies a full-factorial approach to systematically resolve the coupled effects of polymer concentration and pH on the rheological properties of *ETD2020*, with the aim of defining robust formulation regimes tailored for embedded printing. While the resulting data provide a quantitative basis for material selection and matrix design, it is important to note that the printability and structural fidelity in embedded printing also depend on interfacial interactions with the structural ink. These aspects are not experimentally addressed in this work.

## 2. Materials and Methods

### 2.1. Sample Preparation

*Carbopol^®^ ETD2020* (Lubrizol, Wickliffe, OH, USA) was used as a rheological support material and prepared by a controlled thermal mixing protocol. Five polymer concentrations (0.1, 0.3, 0.5, 0.7, and 0.9 wt%) were investigated. The pH was adjusted by adding triethanolamine (TEA, Sigma-Aldrich, St. Louis, MO, USA) in volumetric amounts of 30, 40, or 50 µL per 100 g dispersion, corresponding to target pH values in the range of 6.2–7.5. This pH range was choosen, because it yields stable formulations of *Carbopol^®^ ETD2020* [12,13,20,27]. A pH measurement of the mixed solution was not performed, since the neutralization of *Carbopol^®^* and the ensuing network formation induce a time-dependent drift in pH, making any single-point measurement an unreliable indicator. Furthermore, preliminary point measurements during and after mixing yielded non-reproducible results, preventing a consistent correlation with formulation parameters. The surface tension of *Carbopol^®^* dispersions and therefore the achieved printing fidelity remains effectively unchanged across a wide pH range and is almost similar to purified water, indicating no significant correlation between pH and surface tension [25,28]. The given concentrations represent a wide spectrum relevant within the field of embedded printing [12,20,26,29].

Prior to dispersion, deionized water was preheated to 70 °C and stirred at 550 rpm for 15 min to allow CO_2_ saturation and stabilize the pH at 5.6, which was confirmed using a digital pH meter (Dostmann PH CHECK, Dostmann Electronic GmbH, Wertheim-Reicholzheim, Germany) and validated by pH indicator strips. *Carbopol^®^ ETD2020* powder was sieved to ensure a uniform grain size distribution and slowly added to the heated water. The mixture was stirred at 550 rpm and 70 °C for an additional 45 min to facilitate full dispersion without polymerization. The pH adjustment was then performed by adding TEA using a precision pipette. Following the addition of TEA, the dispersion was subjected to a sequence of homogenization steps: 60 s of stirring at 500 rpm with manual beaker movement, 60 s of manual stirring using a sterilized glass rod, and a final 60 s stirring cycle (500 rpm, 70 °C) to ensure uniform mixing [30]. To remove air inclusions, all samples were degassed under vacuum (100 mbar, 5 min, 3 cycles).

### 2.2. Rheological Measurements

To systematically capture the rheological behavior of *Carbopol^®^ ETD2020* hydrogels, steady-state flow and oscillatory shear measurements were performed. All measurements were conducted using a rheometer (Modular Compact Series, Anton Paar, Seongnam, Republic of Korea) at 20 °C on a parallel-plate geometry (Ø 50 mm) with a plate gap of 0.5 mm. These experiments enabled the quantification of rheological parameters relevant to embedded printing over a broad range of shear rates and network formulations. The full-factorial matrix of 15 conditions (5 polymer concentrations × 3 TEA levels) was evaluated and mapped across key rheological parameters such as $\tau_{y}$, *n*, *k*, η, and $G^{\prime }$.

Steady-state flow behavior was characterized using controlled shear rate experiments using a logarithmic sweep from $200\;s^{-1}$ to $0.001\;s^{-1}$. This range was selected to ensure resolution of both low-shear (rest-like) and high-shear (processing-like) regimes, with the upper limit including the maximum process-relevant shear rate ($\dot{\gamma }\approx 100\;s^{-1}$), calculated for a nozzle diameter of 0.50 mm and a print speed of 50 mm/s [8].

Each measurement was preceded by a pre-shear of $100\;\;s^{-1}$ for 30s, followed by a rest period of 30s to establish uniform starting conditions [19]. To minimize potential artifacts from structural reconstruction or time-dependent effects, the shear ramp was applied in descending order, and all dispersions were confirmed to exhibit negligible thixotropy under these conditions [15,19]. For each composition, three independent repetitions were recorded and averaged [22]. Standard deviations are not reported due to the limited sample volume.

The experimental flow curves (shear stress τ over shear rate $\dot{\gamma }$) were modeled using the nonlinear Herschel–Bulkley equation [19,24]:

$$\tau \left(\dot{\gamma }\right)=\tau_{y}+k\cdot {\dot{\gamma }}^{n}\;\mathrm{for}\;\tau >\tau_{y}$$

where $\tau_{y}$ is the yield stress, *k* the consistency index and *n* the flow index. Parameter fitting was performed using nonlinear regression across the full shear rate range. Goodness-of-fit was evaluated by root mean square error (RMSE), ensuring quantitative validity of the model for all matrix formulations.

To assess viscoelasticity and network formation, oscillatory amplitude sweeps were performed at a constant angular frequency of 1 Hz. These sweeps were conducted in stress-controlled mode by logarithmically increasing the shear stress from 0.1Pa to 300Pa. Storage modulus $G^{\prime }$ and loss modulus $G^{\prime \prime }$ were recorded as a function of increasing shear stress amplitude. The yield point was operationally defined as the crossover of $G^{\prime }$ and $G^{\prime \prime }$ [19]. As in the rotational measurements, each oscillatory test was repeated three times to ensure reproducibility. The results were used to evaluate the deformation resistance and integrity of the internal structure of the gels across the formulation space.

## 3. Results and Discussion

### 3.1. Viscosity Behavior

**Figure 1:**
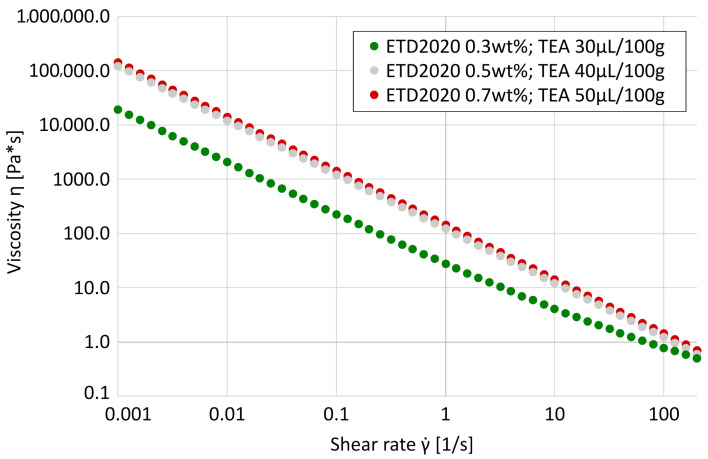
Representative flow curves showing dynamic viscosity η as a function of shear rate $\dot{\gamma }$ for selected *Carbopol^®^ ETD2020* formulations. All samples exhibit pronounced shear-thinning behavior across the tested range ($\dot{\gamma }=0.001$–$200\;s^{-1}$).

All investigated *Carbopol^®^ ETD2020* formulations exhibit pronounced shear-thinning behavior, consistent with previous reports on *Carbopol^®^ Carbomers*. The dynamic viscosity η decreased by several orders of magnitude in the shear rate range ($\dot{\gamma }=0.001$–$200\;s^{-1}$), allowing structural stability at rest and printability under load. The representative flow curves shown in Figure 1 demonstrate a continuous decrease in η with an increase in shear rate. This trend was consistently observed in all 15 formulations.

**Figure 2:**
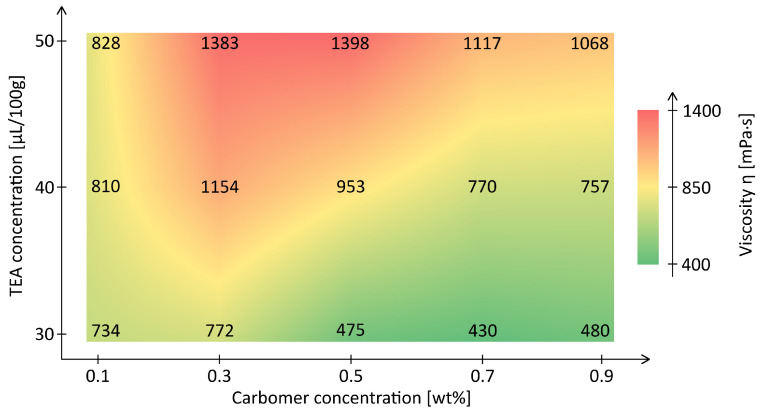
Heatmap of viscosity η for *Carbopol^®^ ETD2020* formulations as a function of polymer concentration and TEA dosage for a shear rate of $\dot{\gamma }=100\;s^{-1}$.

The viscosity is strongly dependent on the polymer concentration and neutralization level as seen in Figure 2. At a typical shear rate in embedded printing of $\dot{\gamma }=100\;s^{-1}$, the sample with 0.7wt% and 30µL TEA reached η≈430mPa·s, while the highest viscosity was observed for 0.5wt%/50µL (η≈1400mPa·s). A comparably high viscosity was measured for the 0.3wt%/50µL formulation. This exceptionally high viscosity is consistent with its low flow index (n≈0.4), suggesting pronounced network formation [29]. These results highlight the tunability of network strength.

### 3.2. Herschel–Bulkley Model Parameters and Fit Quality

**Figure 3:**
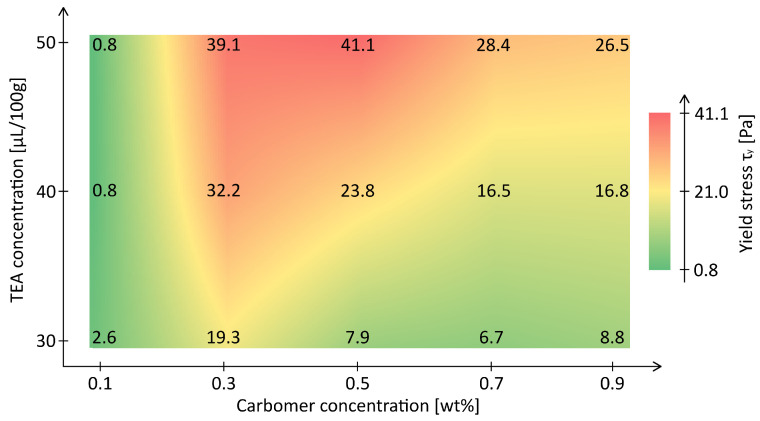
Heatmap of yield stress $\tau_{y}$ for *Carbopol^®^ ETD2020* formulations as a function of polymer concentration and TEA dosage.

The yield stress $\tau_{y}$, determined by Herschel–Bulkley modeling, ranged from below 1Pa at 0.1wt% to above 41Pa at 0.5wt%, demonstrating a strong nonlinear dependence on polymer concentration. Figure 3 visualizes $\tau_{y}$ across the full-factorial design space. Here a strong interconnection between viscosity η and yield stress $\tau_{y}$ is apparent.

At fixed concentrations, increasing the TEA dose consistently increased $\tau_{y}$. For example, $\tau_{y}$ increased from approximately 8Pa to 40Pa between 30µL and 50µL TEA at 0.5wt%. This is attributed to carbomer swelling with increasing pH [22]. Since no plateau is observed, it is likely that a neutral pH as well as a complete network formation have not been reached for lower TEA concentrations [29]. In contrast, the slight increase in yield stress from 0.3wt%/50 µL to 0.5wt%/50 µL suggests that the sample 0.3wt%/50µL lies within such a plateau. This is supported by the materials flow index of n=0.4, characteristic of fully swollen neutralized carbomer networks (see Figure A2) [29].

These trends are in agreement with previous studies on *Carbopol^®^* systems used in embedded printing. For example, O’Bryan et al. reported printable formulations with $\tau_{y}\approx 1$–50Pa for *Carbopol^®^ 980-NF* and *Carbopol^®^ ETD2020*, which aligns well with the present compositions [12].

**Figure A1:**
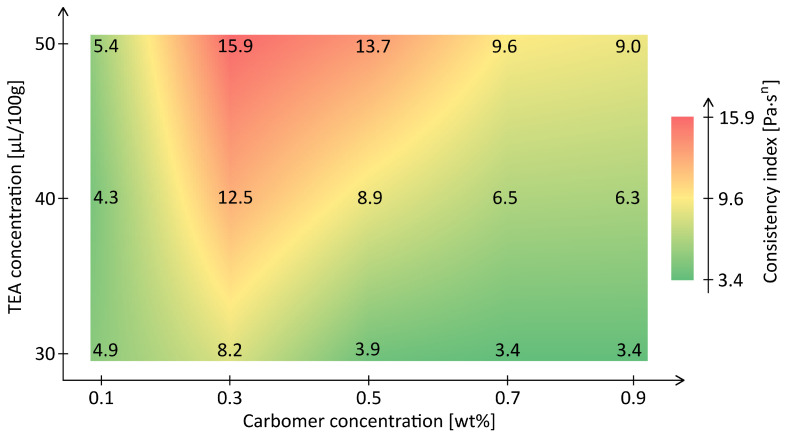
Heatmap of consistency index *k* across formulation matrix. Data extracted from Herschel–Bulkley fits of steady-state flow curves.

**Figure A2:**
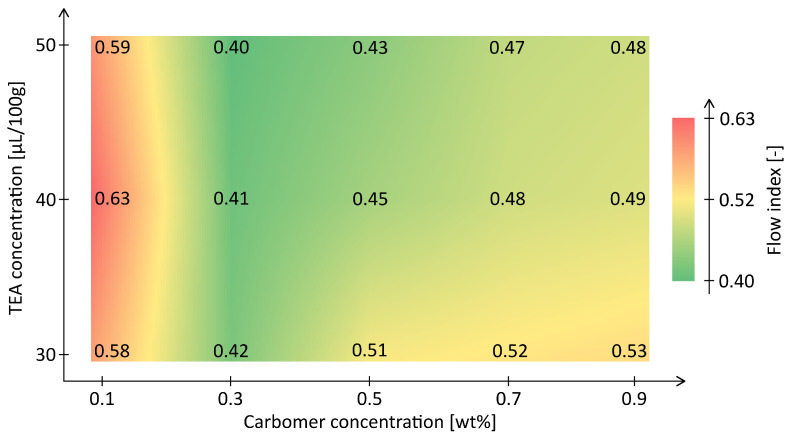
Heatmap of flow index *n* across formulation matrix. Lower *n* values denote stronger shear-thinning behavior.

The consistency index *k* and the flow index *n*, extracted from the Herschel–Bulkley model-fits described in Section 2.2, are shown in Figure A1 and Figure A2 in the Appendix A. Increasing polymer concentration and TEA dosage led to an increase in the consistency index *k*, reflecting enhanced viscosity at low shear rates; the strongest increase was observed at concentrations above 0.5 wt% and TEA additions of up to 40 µL (see Figure A1). Conversely, the flow behavior index *n* decreased with increasing concentration, reaching a minimum of approximately 0.40 and thus indicating pronounced shear-thinning behavior; at a fixed polymer concentration, *n* exhibited a slight increase with higher TEA levels, which indicates incomplete polymerization of the matrix material (see Figure A2).

**Figure 4:**
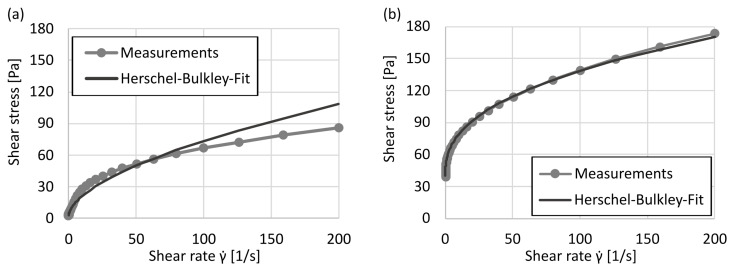
Comparison of experimental flow curves (symbols) and Herschel–Bulkley fits (lines) for two selected *ETD2020* formulations: (**a**) 0.1wt%/30µL; (**b**) 0.3wt%/50µL (right). While the model yields excellent agreement for the 0.3wt% sample (RMSE =0.75), noticeable deviations (RMSE =4.81) occur for low-concentration formulations due to reduced structural coherence and low yield stress.

To assess the quantitative validity of the Herschel–Bulkley model, the experimental and fitted flow curves were compared in all 15 formulations. Representative results are shown in Figure 4. While the model yields excellent agreement for most compositions (RMSE <1.0) as exemplary shown for the 0.3wt%/50µL sample well described by the model (RMSE = 0.75), noticeable deviations occur for weakly structured samples, particularly at low polymer concentrations. The formulations with 0.1wt% show increased residuals in the shear range and a RMSE >4.81. This suggests that the Herschel–Bulkley model is not applicable to weakly structured gels. Although no indications of wall slip (e.g., “dog-leg” flow curves) were observed using a plate gap of 0.5mm, the results for the 0.1wt% formulations should be interpreted with caution.

### 3.3. Storage and Loss Moduli

**Figure 5:**
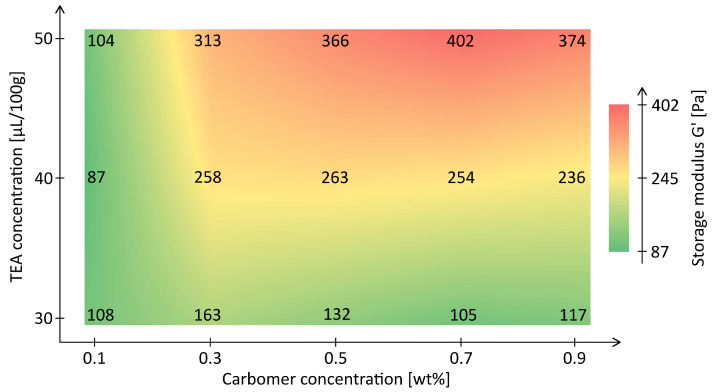
Heatmap of storage modulus $G^{\prime }$ across the *Carbopol^®^ ETD2020* formulation matrix. Higher values indicate increased network stiffness.

Oscillatory amplitude sweeps at 1Hz were used to assess viscoelasticity and network stability. All formulations exhibited an elastic-dominated regime at low strain ($G^{\prime }>G^{\prime \prime }$), confirming solid-like behavior at rest. Both polymer concentration and TEA dosage increased $G^{\prime }$, with maximum values in the range of ≈400Pa for 0.9wt%/(50µL). In contrast, weakly structured formulations (e.g., 0.1wt%/30 µL) showed a storage modulus of $G^{\prime }\approx 100\;\mathrm{Pa}$. Figure 5 shows the heatmap of $G^{\prime }$ across the formulation matrix.

A strong dependence on polymer concentration is evident, with TEA exerting an additional effect especially between 0.3 and 0.7wt%. A coherent gel structure with reproducible elasticity emerges from approximately 0.3wt% upward, provided sufficient TEA is present.

The yield point, defined by the $G^{\prime }$/$G^{\prime \prime }$ crossover, shifted to higher stress levels with increasing concentration and pH, in line with trends in yield stress $\tau_{y}$. Several mid-to-high concentration formulations exhibited a stable elastic plateau, indicating robust gel networks suitable for embedded printing. Low-concentration samples lacked a pronounced linear viscoelastic region and yielded at low amplitudes.

### 3.4. Time Dependency of Rheological Properties

**Figure A3:**
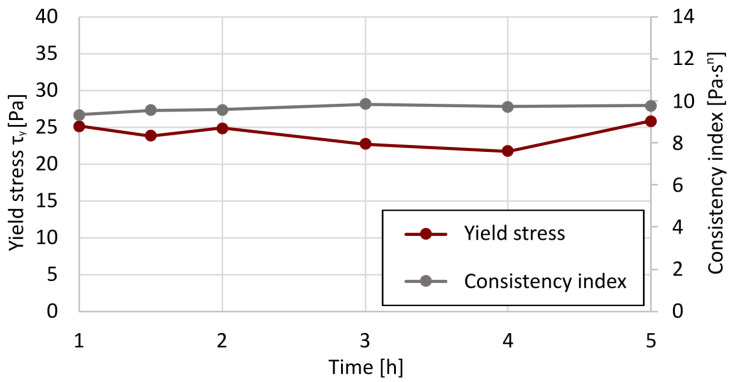
Time dependency of yield stress $\tau_{y}$ and consistency index *k* over a duration of 5 h. No noticeable change was measured.

With increasing Carbomer concentration, the dispersion pH decreases, leading to a reduction in yield stress at constant TEA levels due to incomplete polymerization. This interpretation is further supported by the observed increase in turbidity for higher-concentration matrices, which likewise indicates incomplete network formation. Consequently, rheological properties may evolve over time. For the 0.9wt%/50 µL formulation, three rotational measurements performed hourly over the first five hours post-preparation showed no significant change in yield stress or consistency index, thus defining this interval as the processing window of constant rheological behavior (see Figure A3). To assess delayed polymerization beyond this window, the same sample was re-examined after five days of airtight storage; the yield stress had increased by approximately 10 Pa, confirming time-dependent network development.

## 4. Conclusions

This study analyzes the rheological parameters for *Carbopol^®^ ETD2020* as a support matrix for embedded printing by systematically evaluating a full-factorial range of polymer concentrations (0.1–0.9wt%) and neutralization levels (30–50µL TEA per 100g).

### 4.1. General Trends and Model Accuracy

All *Carbopol^®^ ETD2020* formulations exhibited pronounced shear-thinning behavior across the full range of tested shear rates ($\dot{\gamma }=0.001$–$200\;s^{-1}$), consistent with expectations for crosslinked polyacrylic acid hydrogels. Both the dynamic viscosity and the yield stress increased strongly with polymer concentration and TEA dosage, reflecting the formation of progressively denser and more elastic networks. This trend aligns with expected swelling behavior of carbomer microgels. Notably, the effects of polymer content and TEA concentration are not independent but act in a synergistic manner: sufficient TEA is required to unlock the full swelling capacity of the polymer network, particularly at intermediate concentrations (e.g., 0.3–0.5wt%).

The Herschel–Bulkley model provided excellent fits for nearly all formulations, with RMSE values < 1.0. However, a clear breakdown of model fidelity was observed for 0.1 wt% compositions, which showed significantly higher residuals and poor agreement between experimental and fitted flow curves (RMSE >4.8). This is explained by the lack of a coherent network structure and the absence of a true yield point in these weakly structured gels. In such cases, the flow behavior transitions gradually from elastic to viscous with no distinct yield threshold, making the application of a yield-stress model conceptually inappropriate.

From a processing standpoint, these low-concentration formulations are not viable as support matrices for embedded printing. Their insufficient yield stress and low viscosity under rest conditions prevent stabilization and promote unwanted flow or diffusion around the nozzle path. In contrast, formulations at or above 0.3 wt% with sufficient TEA display well-defined yield behavior and reproducible rheological profiles, indicating structurally stable networks suitable for embedded extrusion. This supports the need for both adequate polymer content and neutralization level to ensure robust matrix performance. Further investigations should define the cut-off point of usable polymer concentrations for <0.3 wt%.

### 4.2. Design Window and Suitability for Printing

The full-factorial data set reveals a clear rheological design space suitable for embedded printing applications. Formulations characterized by a yield stress between 6.7 and 41.1Pa, a consistency index *k* between 3.4 and 15.9, and a flow index *n* in the range of 0.40 to 0.53 exhibit stable, shear-thinning behavior under printing-relevant conditions. The lower bound of $\tau_{y}$ defines the threshold required to resist buoyancy-driven displacement and material tear-off, while upper bounds correlate with increasing network rigidity that may impede nozzle motion, and increase cavity formations.

Within this window, the flow index *n* emerges as a critical indicator of matrix quality. Formulations approaching n≈0.40 consistently coincide with fully neutralized carbomer networks, as indicated by high $G^{\prime }$ values and plateauing $\tau_{y}$ behavior. Conversely, samples with n>0.45 show signs of incomplete polymerization and progressive softening under shear, suggesting residual unneutralized carbomer clusters. These clusters may accumulate at the matrix-ink interface during extrusion, potentially degrading surface fidelity and resolution. While direct interfacial effects were not measured in this study, we hypothesize that insufficiently neutralized matrices lead to reduced feature quality in embedded printing due to poor matrix cohesion at the ink boundary.

The interplay of $\tau_{y}$, *k*, and *n* thus governs both the structural stability of the matrix and its printability. High *k* values ensure resistance to deformation at low shear, whereas low *n* values support facile shear-induced displacement during nozzle passage. Formulations with both high $\tau_{y}$ and low *n*, particularly at 0.3wt% and 50 µL TEA, define an optimal zone for matrix-supported freeform fabrication. These findings provide a quantitative foundation for selecting *ETD2020* formulations tailored to specific process requirements.

### 4.3. Network Formation and Elasticity

Oscillatory shear measurements further substantiate the structural integrity of the investigated formulations. At low stress amplitudes, all samples with ≥0.3wt% polymer concentration and sufficient TEA dosage exhibited elastic-dominated behavior ($G^{\prime }\gg G^{\prime \prime }$), indicating the presence of stable, percolated networks. The storage modulus $G^{\prime }$ increased with both concentration and TEA volume, reaching values above 400Pa for the 0.7wt%/50 µL formulation.

The evolution of $G^{\prime }$ across the formulation matrix mirrors trends observed in $\tau_{y}$, reinforcing its role as a complementary indicator of matrix strength. Importantly, the elastic plateau observed in mid-to-high concentration systems denotes deformation-resistant gels capable of supporting features during printing.

### 4.4. Limitations and Future Work

While the present study defines a robust rheological design window for *Carbopol^®^ ETD2020* based on concentration and neutralization level, limitations must be acknowledged. Most notably, the influence of matrix–ink interfacial interactions was not experimentally investigated. However, the observed increase in flow index *n* above 0.40 at insufficient TEA levels suggests incomplete polymerization, which may result in heterogeneous microgel domains near the interface. We hypothesize that such local heterogeneities negatively affect strand definition, surface quality, and material fusion during embedded printing. Verifying this assumption requires dedicated interfacial characterization at the matrix boundary. Future studies should therefore focus on quantifying interfacial compatibility with representative inks as well as evaluating formulation robustness under realistic processing conditions.

## Data Availability

All raw data is freely available under [31].
